# New insights and predictability from in vivo recordings of paroxysmal sympathetic hyperactivity in disorders of consciousness

**DOI:** 10.1007/s10286-025-01175-z

**Published:** 2025-12-24

**Authors:** Francesco Riganello, Maria D. Cortese, Martina Vatrano, Lucia F. Lucca, Maria E. Pugliese, Maria Ursino, Elio Leto, Antonio Cerasa, Nicholas Schiff, Andrea Soddu

**Affiliations:** 1 Reseach in Advanced NeuroRehabilitation, S. Anna Institute, 88900 Crotone, Crotone Italy; 2 Institute for Biomedical Research and Innovation (IRIB.), National Research Council of Italy (CNR), 98100 Messina, Italy; 3 Department of Neurology and Neuroscience, Weill Cornell Medical College, New York, NY USA; 4 Physics and Astronomy Department, Western Institute for Neuroscience, University of Western Ontario, London, ON Canada

**Keywords:** Paroxysmal sympathetic hyperactivity, Heart rate variability, Autonomic nervous system, Renin–angiotensin–aldosterone system, Support vector machine

## Abstract

**Purpose:**

Paroxysmal sympathetic hyperactivity (PSH) is a severe complication of acquired brain injuries (ABIs), characterized by sudden autonomic surges that exacerbate clinical outcomes. Its pathophysiology remains debated, and early biomarkers are lacking. This study aims to investigate autonomic changes preceding PSH and assess the feasibility of predictive modeling using heart rate variability (HRV).

**Methods:**

Continuous electrocardiogram (ECG) recordings were obtained from six male patients with disorders of consciousness (DoC), including unresponsive wakefulness syndrome and minimally conscious state. A total of 24 PSH episodes and 24 matched control (noPSH) events were analyzed. HRV metrics, including entropy measures and power spectral density (PSD), were evaluated. A support vector machine (SVM) classifier was implemented to differentiate PSH from control events and to predict PSH onset.

**Results:**

PSH events were associated with significant heart rate increases, reduced entropy-based complexity, and decreased PSD in both low-frequency (LF) and high-frequency (HF) bands. An increased very-low-frequency (VLF)/(LF + HF) ratio suggested potential involvement of the renin–angiotensin–aldosterone system (RAAS) in PSH pathogenesis. The SVM classifier achieved perfect classification during the event. In addition, 10 min prior to onset, the model reached 67% sensitivity, 100% specificity, and 83% balanced accuracy.

**Conclusions:**

HRV analysis reveals distinct autonomic signatures preceding PSH and suggests, as a working hypothesis, that dysregulation of the RAAS may play a role. However, VLF power is influenced by multiple mechanisms and cannot be considered a specific or exclusive marker of RAAS activity. SVM-based predictive modeling offers a promising tool for PSH detection, providing a basis for investigating autonomic/neuroendocrine regulation, including RAAS.

**Supplementary Information:**

The online version contains supplementary material available at 10.1007/s10286-025-01175-z.

## Introduction

Acquired brain injuries (ABIs) cause a range of severe complications, notably paroxysmal sympathetic hyperactivity (PSH), which is marked by dramatic surges in autonomic responses such as blood pressure, heart rate, and muscle rigidity, significantly challenging clinical management [1]. Affecting 8–33% of patients with ABI in the acute phase [1], PSH exacerbates outcomes through hyperthermia, catabolism, and spasticity [2, 3], highlighting the urgent need for a deeper understanding and more innovative management strategies. Recent analyses reveal a diversified occurrence of PSH across various etiologies, suggesting that a wide spectrum of brain injuries can trigger PSH and underscoring the importance of vigilant diagnosis across all ABI forms [4, 5].

The pathophysiology of PSH, still under active investigation, encompasses theories from the disconnection hypothesis, which suggests a dissociation between cortical inhibitory centers and sympathetic control areas [1], to the excitatory/inhibitory ratio model that points to an imbalance in neuronal activities leading to enhanced sympathetic output [1]. Additional factors, such as neuroendocrine responses [6] and the role of neutrophil extracellular traps (NETs) in sympathetic excitation [7], further complicate our understanding of PSH.

Despite extensive case reports and studies on PSH [5, 8], a notable gap persists in our understanding of the autonomic changes preceding and during PSH crises, primarily attributed to their unpredictability. This unpredictability hinders the systematic analysis of autonomic responses.

Heart rate variability (HRV) has emerged as a pivotal tool in this investigation, offering noninvasive insight into the autonomic nervous system’s (ANS) dynamics by measuring intervals between heartbeats [9]. HRV extends its utility beyond cardiovascular monitoring, shedding light on the nervous system’s adaptive responses, including nociceptive processing [10] and a broad range of autonomic functions, as evidenced in patients with ABI [11]. By employing HRV analysis, this study endeavors to uncover the autonomic disturbances characterizing PSH, potentially leading to the identification of predictive markers.

This study takes a novel approach by comparing PSH episodes with noPSH events (characterized by increased heart rate) and analyzing autonomic conditions preceding these events in patients with disorders of consciousness (DoC). DoC includes unresponsive wakefulness syndrome (UWS) [12], characterized by spontaneous eye-opening and sleep–wake cycles without signs of awareness or purposeful behaviors, and minimally conscious state (MCS) [13], in which patients show inconsistent yet clear signs of minimal awareness and purposeful responses to stimuli. Patients with MCS are further categorized into MCS minus and MCS plus, classified using the Coma Recovery Scale-Revised (CRS-R) [13].

This comparative analysis aims to identify distinct cardiovascular and autonomic nervous system (ANS) markers characterizing each state. It represents the first detailed HRV analysis of PSH events within a clinical framework. In this study, we also explored, as a working hypothesis, whether alterations in very-low-frequency (VLF) power, a multifactorial measure that reflects several slower-acting physiological mechanisms, could be indirectly consistent with renin–angiotensin–aldosterone system (RAAS) dysregulation. In addition, a support vector machine (SVM) classification model was employed to leverage its robust pattern recognition capabilities in differentiating PSH from noPSH events. By examining autonomic changes preceding and during PSH episodes and integrating HRV metrics with machine learning techniques, this study seeks to enhance the understanding of autonomic regulation and establish new benchmarks for utilizing HRV as a diagnostic and analytical tool in managing these complex neurological conditions.

## Materials and methods

### Patient selection

Patients exhibiting PSH were identified by the PSH-Assessment Measure (PSH-AM), which encompasses the Clinical Features Scale (CFS) and the Diagnosis Likelihood Tool (DLT) [14] (Supplementary Material: Supplementary Fig. S2). The CFS evaluates six domains—heart rate, respiratory rate, systolic blood pressure, temperature, sweating, and posture—with severity scores ranging from 0 (absent) to 3 (severe). A cumulative score above 13 signals severe conditions. The DLT assesses 11 factors, including the co-occurrence of clinical features, the paroxysmal nature of episodes, and their daily frequency, yielding a score from 0 to 11. A combined PSH-AM score of 19–21 suggests a probable PSH event.

### Patient descriptions

Six male patients (age 27 ± 14 years) were admitted to the semi-intensive care unit following stabilization in intensive care, presenting with generalized hypertonia (Table 1). In one patient, the etiology included cardiac arrest and two hemorrhagic and three traumatic brain injuries. Five of them had been diagnosed with UWS, and one had MCS. During PSH episodes, all patients showed increases in blood pressure, sweating, heart rate, and hypertonia. To control PSH events, all patients received β-blocker therapy (propranolol or bisoprolol) and antiepileptic prophylaxis (levetiracetam and/or phenobarbital). In addition, antihypertensive agents (clonidine, amlodipine) were administered during the period of interest surrounding PSH episodes. At 3 months, three patients with UWS had a change in diagnosis in MCS; one patient emerged from MCS, while two continued to exhibit UWS.

**Table 1:** Demographic information

Diagnosis	Etiology	Sex	Age	Time from injury (days)	Recorded PSH	CRS-R at hospitalization	Diagnosis at 3 months	CRS-R at 3 months	Trauma description
UWS	CA	Male	16–55	52 ± 30	2	7	UWS	6	Bilateral hypodensity in the basal ganglia, more pronounced on the left side, as well as in the splenium of the corpus callosum and the midbrain, resulting from ischemic changes
	HEM				2	4	MCS	12	Expansive lesion in the right occipital region. Intra-axial hemorrhage with perilesional edema and mass effect on the ventricular system. Hypodensity of the body of the corpus callosum and brainstem
					9	7	UWS	5	Intra-axial hemorrhagic focus was reported in the occipital-mesial area. In the posterior cingulate gyrus, perilesional edema was found with a mass effect on the right ventriculus and an initial dilatation of the same on the temporal corn
	TBI				4	6	MCS	12	Extracranial soft tissue swelling in the right fronto-temporo-parietal region. Multiple lacerative-contusional and hemorrhagic foci in the posterior pontomesencephalic area (predominantly left), right thalamic-capsular region, left anterior capsular nucleus, splenium of the corpus callosum, bilateral hippocampal regions, and the right temporomesial and temporopolar areas. Hemorrhages were observed in the right interpeduncular and perimesencephalic cisterns, the aqueduct, the occipital horn of the right lateral ventricle, and the third ventricle
					6	6	MCS	12	Cerebral contusion; petechial hemorrhages in the right temporal and occipital area and frontal bilateral and frontal areas; and minimal number of subarachnoid hemorrhages. Multiple lacerate-contusive temporal–occipital lesions on the right and bilateral frontal and minimal residues of subarachnoid hemorrhage. Signal changes with hyperintensity in the T2 fluid-attenuated inversion recovery (FLAIR) sequences in the midbrain, in the cerebral peduncles, and in the ventral nuclei of the thalami bilaterally were also reported. The same sequence detected a hyperintensity focus in the corpus callosum’s splenium posteriorly. Axonal and deep subcortical damage was also observed
MCS					1	12	EMS	20	Large right parieto-occipital epidural hematoma causing effacement of the sulci, compression of the ipsilateral lateral ventricle, and a midline shift to the left of about 8 mm

*UWS* unresponsive wakefulness syndrome, *MCS* minimally conscious state, *EMS* emerged from MCS, *m* male, *TBI* traumatic brain injury, *HEM* hemorrhagic, *CA* cardiac arrest

### Data acquisition

A total of 48 events (24 PSH and 24 noPSH) were recorded using the BioPatch device (Zephyr Technology). The BioPatch is a Food and Drug Administration (FDA)-approved, continuous electrocardiogram (ECG) monitoring system that captures one-lead ECG data at a sampling rate of 250 Hz, with an input amplitude range of 0.25–15 mV. The device was affixed to the chest using two adhesive patches. A key feature of the BioPatch system is the “heart rate confidence” metric, which evaluates the quality of the ECG signal on the basis of the signal-to-noise ratio. Owing to the device’s advanced filtering and amplification circuitry, it is possible to ensure reliable heart rate data even during patient movements.

ECG signals were continuously logged and stored on the device’s flash memory. Monitoring was conducted for at least 8 h daily, starting after morning clinical assessments and routine care activities to minimize potential disturbances and artifacts in the ECG recordings.

The data analysis workflow involved the following steps: (i) medical staff annotated the onset time of PSH events on the basis of clinical observations; (ii) for each annotated event, ECG data were extracted from two distinct time windows: 20 min before the event onset divided into two adjacent blocks of 10 min (Pre_20_ and Pre_10_) and event: 10 min after the event onset.

Each window comprised 10 min of continuous ECG data, necessary to capture a sufficient number of very-low-frequency (VLF) oscillations, which are essential for robust and reliable analysis of autonomic function within this frequency band.

Data integrity and processing: Extracted ECG data were inspected for signal integrity to ensure the absence of artifacts or ectopic beats. Subsequently, the data were processed for heart rate variability (HRV) analysis.

### Physiological context

Respiratory rate (breaths/min) was estimated from ECG-derived respiration (EDR) using Kubios HRV (version 3.1; Kuopio, Finland). Noninvasive blood pressure (mmHg) was annotated by medical staff from bedside monitors at the pre-event window and during the event. Both variables were summarized descriptively (mean ± standard deviation [SD], median [IQR], min–max) per condition (PSH and noPSH).

### HRV analysis

In analyzing the ECG data for patients diagnosed with PSH, the Kubios HRV advanced software (version 3.1, Kuopio, Finland) was used, allowing a detailed examination across time, frequency, and nonlinear domains [15]. The preprocessing phase included a rigorous noise assessment, followed by detecting R peaks using Kubios’s adaptation of the Pan–Tompkins algorithm, a choice adopted for its proven effectiveness in QRS detection [16]. To refine R-peak identification and address potential artifacts or ectopic beats, we applied a 4 Hz cubic spline interpolation, supplemented by a subsequent visual inspection for any necessary manual adjustments.

A quadratic polynomial model was employed to detrend the RR interval series, aiming to mitigate the influence of lower-frequency oscillations on the power spectral density (PSD) analysis to accommodate the nonstationary nature of biological signals. We calculated the PSD using the Fast Fourier Transform method, specifically applying Welch’s method with a window width of 150 s. This approach was selected to derive the natural logarithms of high frequency (HF: 0.15–0.5 Hz) (LnHF), low frequency (LF: 0.04–0.15 Hz) (LnLF), and very low frequency (VLF: 0.0033–0.04 Hz) (LnVLF), as well as the LF/HF ratio, ensuring an accurate representation of the HRV spectral components. The logarithmic transformation addressed the measures’ skewed distribution, with skewness values ranging between 2.5 and 5.4, thereby normalizing the distribution for further analysis.

The multiscale entropy (MSE) analysis quantifies the signal’s nonlinear and nonstationary attributes over varying time scales [17]. The signal’s irregularity was assessed by averaging RR intervals within nonoverlapping windows of increasing lengths (1–10) and applying sample entropy (SampEn) [18] to these coarse-grained series. Setting the dimensional phase space *m* = 2 and the matching tolerance *r* = 0.2 facilitated robust complexity measurements across diverse data lengths.

MSE analysis provided short- and long-scale complexity indices (CI_s_ and CI_l_), calculated as the sum of SampEn values across coarse-grained scales 1–5 and 6–10, respectively. These indices provide insights into the dynamics of autonomic nervous system regulation over relevant temporal frameworks, offering critical information about the sympathetic and parasympathetic activity involved in PSH [17].

### Wavelet analysis and linear trend

Wavelet analysis (MATLAB’s Wavelet Toolbox [version 2024a]) was employed to characterize temporal changes in the VLF, LF, and HF bands and their relative ratios (e.g., VLF/[LF + HF]). Compared with traditional Fourier-based methods, wavelet analysis provides enhanced temporal and frequency resolution, making it particularly suitable for capturing transient and localized variations in HRV. The Morlet wavelet was selected owing to its optimal balance between time and frequency localization, which is essential for accurately identifying the VLF, LF, and HF oscillations within our ECG recordings.

The ECG data were interpolated at 4 Hz before applying the wavelet transform. This interpolation rate was chosen to sample the VLF band, adequately preventing aliasing artifacts. The interpolation process employed a cubic spline method, which maintains the smoothness and continuity of the RR interval series while accommodating the low-frequency components essential for our study.

After the wavelet analysis, the linear trend analysis was performed to account for any gradual changes in the RR interval series over time. This analysis provided the linear equations (intercept and time coefficient), the standard error of the coefficient, the *F*-ratio, the *p*-values, the 95% confidence interval for the time coefficient, and the *R*^2^ value.

### Statistical analysis

For the between-condition analysis, PSH and noPSH events were compared using independent samples *t*-tests to evaluate differences in HRV parameters. In the within-condition analysis, paired samples *t*-tests were used to compare data recorded during the event, 10 min prior (Pre_10_), and 20 min prior (Pre_20_). Before conducting *t*-tests, data were assessed for normality using the Shapiro–Wilk test. The Bonferroni correction was applied to control the type I error rate, with the significance threshold set at *p* = 0.001, corresponding to the Bonferroni-adjusted alpha (0.05/44) for the maximum number of pair-wise comparisons.

### Support vector machine

The SVM was selected for its robustness in handling small datasets and superior performance in nonlinear classification tasks, making it well-suited for the HRV-based PSH prediction model.

Our study used JMP software (version 16, SAS Institute, Cary, NC, USA) to implement a support vector machine (SVM) model to differentiate between PSH and noPSH events. The SVM approach is particularly advantageous for complex datasets as it effectively identifies the optimal boundary that maximizes the separation between groups, mitigating the risk of overfitting while ensuring generalizability to new data.

The model’s efficacy was enhanced by employing the radial basis function (RBF) kernel, simplifying data categorization by transforming the input data into a more distinguishable format. This combination of SVM with the RBF kernel allows for a nuanced model that is both flexible and accurate, adept at navigating the intricate relationships in the data.

Crucially, the model’s performance hinges on carefully calibrating the cost (*C*) and RBF gamma parameters. The *C* parameter balances the margin maximization with classification error minimization, while the gamma parameter modulates the decision boundary’s adaptability. Proper tuning of these parameters is vital for optimizing the model’s complexity and its ability to perform accurately on unseen datasets.

The tenfold cross-validation was used to evaluate the model’s predictive power comprehensively. It divides the data into ten parts, ensuring each subset accounts for about 10% of the data. This approach safeguards against overfitting and enhances the reliability of our findings by providing a robust evaluation of the model across multiple training and validation scenarios, minimizing bias, and validating the model’s capacity to generalize well to new, unseen data, ensuring an accurate and reliable assessment of its performance.

## Results

### Physiological context

EDR-derived respiratory rates remained within a normal range with no extreme bradypnea (< 10 breaths/min). Distributions overlapped across windows within both conditions, with no systematic shift between PSH and noPSH or across windows. In PSH, mean ± SD rates were 19 ± 5 breaths/min at Pre_20_ and Pre_10_, and 21 ± 4 at the event. In noPSH, rates were 18 ± 6 at Pre_20_ and 19 ± 6 at Pre_10_ and event. All observed respiratory rates fell within the predefined HF band used for spectral analysis, ensuring that high-frequency power was not underestimated. Summary values are reported in Supplementary Table S1.

PSH recordings showed a clear descriptive rise in systolic/diastolic blood pressure at the event compared with the pre-event window, whereas noPSH showed modest changes. Specifically, in PSH, blood pressure (BP: systolic mean ± SD / diastolic mean ± SD) rose from 136 ± 9/82 ± 10 mmHg (pre-event) to 183 ± 5/92 ± 8 mmHg (event), i.e., a mean +47 mmHg systolic. In noPSH, BP changed from 115 ± 7/69 ± 7 mmHg to 125 ± 8/73 ± 5 mmHg, i.e., a mean +10 mmHg systolic. Details are provided in Supplementary Table S2.

### Heart rate variability analysis

Tachogram analysis showed a significant escalation in mean heart rate (HR) approaching PSH episodes, contrasting sharply with noPSH events. Specifically, we observed a significant HR increase from an average of 97 ± 14 beats per minute (b/min) (max HR 116 ± 18 b/min) 20 min before an event (Pre_20_) to 105 ± 15 b/min (max HR 120 ± 16 b/min) 10 minutes prior (Pre_10_) (*t*-test: *t*(23), *t* = −4.69; *p* = 0.0001; *r*^2^ = 0.49), peaking at 123 ± 12 b/min (max HR 138 ± 10 b/min) during the PSH episode, with a significant difference comparing Pre_10_ versus event condition (mean HR, *t*-test: *t*(23), *t* = −7.32; *p* = 0.0001; *r*^2^ = 0.70; and max HR, *t*-test: *t*(23), *t* = −6.92; *p* = 0.0001; *r*^2^ = 0.67). In contrast, under noPSH conditions, we observed only a slight increase in HR across the three time windows (Pre_20_: mean HR 95 ± 13 b/min [max 107 ± 11]; Pre_10_: 98 ± 14 b/min [max 109 ± 14]; event: 100 ± 13 b/min [max 111 ± 14]) without significant differences among them. Comparing PSH and noPSH for mean and max HR, the differences were significant during the event (mean HR, *t*-test: *t*(46), *t* = −6.08; *p* = 0.0001; *r*^2^ = 0.46; and max HR, *t*(46), *t* = −7.65; *p* = 0.0001; *r*^2^ = 0.56).

**Fig. 1:**
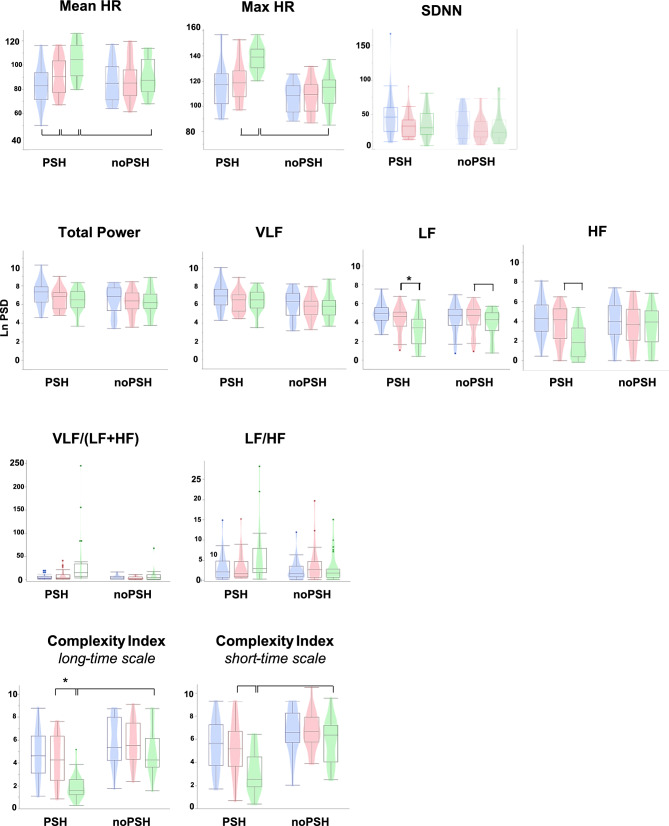
Boxplot and violin plot of time, frequency, and nonlinear domain. PSH and noPSH show paroxysmal sympathetic hyperactivity and significant increases in HR, respectively. In blue, 20 min before the event (Pre_20_); in red, 10 min before the event (Pre_10_); in green, the event. From top to bottom: first line—time domain measure (mean and max heart rate and cardiac variability SDNN); second line—frequency domain measures (natural logarithms of the power spectrum density for total power, very low frequency [VLF], low frequency [LF], and high frequency [HF]); third line—frequency ratios; fourth line—nonlinear domain (complexity index in short-time and long-time scales). Horizontal square brackets indicate significant statistics at 0.0001, with “*” at 0.0003

No significant differences were detected for the standard deviation of NN intervals (SDNN—i.e., RR intervals, free from ectopic beats and artifact RR intervals) (Fig. 1).

### Entropy analysis

The entropy analysis quantifies the unpredictability or irregularity of heart rate fluctuations, providing a measure of the complexity of autonomic control. The complexity index (CI) integrates multiscale entropy values to assess the overall complexity of heart rate dynamics across short- and long-time scales, reflecting scales with faster and slower regulatory processes, respectively. Significantly decreased entropy was observed comparing the event with the previous 10 min (Pre_10_) for CI_s_ (*t*(23): *t* = −5.32, *p* = 0.0001, *r*^2^ = 0.38) and CI_l_ (*t*(23): *t* = −4.02, *p* = 0.0003, *r*^2^ = 0.26). Moreover, CI_s_ and CI_l_ were significantly lower during PSH compared with noPSH during the event (CI_s_ [*t*(46): *t* = −6.81, *p* = 0.0001, *r*^2^ = 0.50]; CI_l_ [*t*(46): *t* = −5.26, *p* = 0.0001, *r*^2^ = 0.38]) (Fig. 1).

### Power spectral density (PSD) analysis

PSD analysis quantifies the variance within different heart rate signal frequency bands, showing how various physiological mechanisms contribute to heart rate fluctuations over time. The PSD in the very-low-frequency (VLF) range (0.0033–0.04 Hz, influenced by slower-acting regulatory mechanisms), low-frequency (LF) range (0.04–0.15 Hz, associated with both sympathetic and parasympathetic activity), and high-frequency (HF) range (0.15–0.5 Hz, primarily related to parasympathetic activity) components was analyzed.

**Fig. 2:**
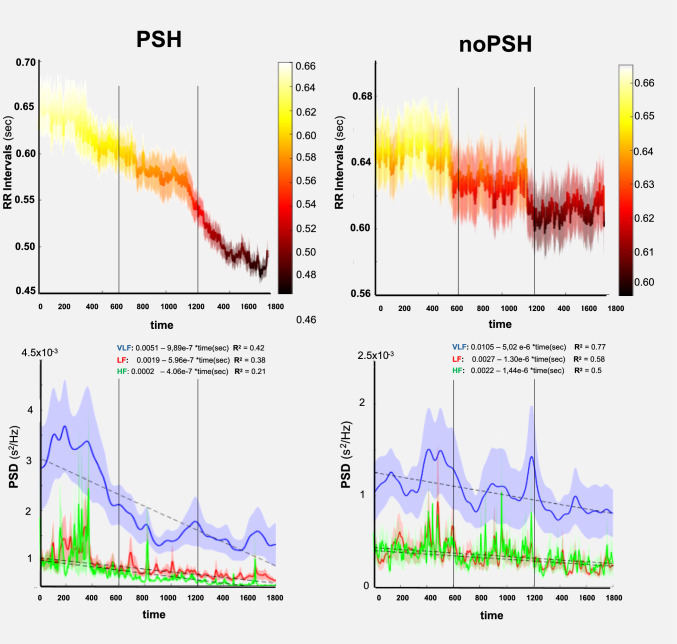
Tachogram and wavelet frequency analysis. Columns on the left and right are the PSH and noPSH, respectively. The vertical dashed lines represent the division of 1800 s (30 min) into three equal blocks of 600 s (10 min.) Top graphs: the tachogram (RR inter-beat in seconds). On the *y*-axis is the RR interval (s) with standard error (SE), and on the *x*-axis is the time in seconds. Colors from light yellow to dark red indicate decreased RR interval corresponding to increased HR (i.e., RR = 0.47 → HR = 85.7; RR = 0.45 → HR = 133.3); the transparent area is the SE. Bottom graphs: the wavelet of the power spectrum density (PSD) in s^2^/Hz with the SE (transparent area) and linear trend (dashed line) for the very low frequency (VLF) in blue, low frequency (LF) in red, and high frequency (HF) in green. On the graph are the linear equation and the relative linear fit *R*^2^

All spectral components exhibited a significant decrease in PSD over time in both PSH and noPSH conditions, with a more pronounced decline observed during PSH events (Fig. 2; Table 2). Specifically, the negative time coefficients for VLF, LF, and HF components were higher in the PSH condition, suggesting an abrupt reduction in autonomic variability.

**Table 2:** Regression analysis of PSD components over time for PSH and noPSH conditions

Component	Condition	Intercept	SE intercept	Time coefficient (per second)	SE time coefficient	*F*-ratio	*p*-Value	*R*^2^	95% confidence interval for time coefficient
VLF	PSH	0.0105	6.78 × 10^−5^	−5.02 × 10^−6^	6.52 × 10^−8^	5920.22	< 0.0001	0.77	[−5.14 × 10^−6^, −4.90 × 10^−6^]
	noPSH	0.0051	2.88 × 10^−5^	−9.89 × 10^−7^	2.77 × 10^−8^	1276.10	< 0.0001	0.42	[−1.03 × 10^−6^, −9.56 × 10^−7^]
LF	PSH	0.0027	2.70 × 10^−5^	−1.30 × 10^−6^	2.60 × 10^−8^	2478.60	< 0.0001	0.58	[−1.36 × 10^−6^, −1.24 × 10^−6^]
	noPSH	0.0019	1.88 × 10^−5^	−5.96 × 10^−7^	6.67 × 10^−8^	1080.30	< 0.0001	0.38	[−6.29 × 10^−7^, −5.63 × 10^−7^]
HF	PSH	0.0022	3.50 × 10^−5^	−1.44 × 10^−6^	3.37 × 10^−8^	1829.80	< 0.0001	0.50	[−1.51 × 10^−6^, −1.37 × 10^−6^]
	noPSH	0.0002	1.90 × 10^−5^	−4.06 × 10^−7^	1.83 × 10^−8^	489.30	< 0.0001	0.21	[−4.42 × 10^−7^, −3.70 × 10^−7^]

Intercept: estimated PSD value at time zero; time coefficient: estimated change in PSD per second; SE: standard error of the coefficient; *F*-ratio: ratio used in analysis of variance (ANOVA) to determine the overall significance of the model; *p*-value: indicates the statistical significance of the coefficient; *R*^2^: coefficient of determination, representing the proportion of variance explained by the model; 95% confidence interval for time coefficient: confidence interval for the time coefficient, providing a range of values within which the true coefficient is likely to fall

*PSH* paroxysmal sympathetic hyperactivity condition, *noPSH* no PSH condition

The observed trend shows a PSH condition characterized by a rapid decline in overall autonomic activity, particularly in parasympathetic modulation (as evidenced by decreased HF power), coupled with an increased dominance of slower regulatory mechanisms reflected in the VLF component (Fig. 2; Supplementary Material: Supplementary Fig. S3 and Supplementary Table S3).

Analysis of Pre_20_, Pre_10_, and the event condition showed significantly lower LF and HF component values during the event condition compared with the preceding phase (Pre_10_) (LF: *t*(23) = −4.23, *p* = 0.0003, *r*^2^ = 0.28; HF: *t* = −5.17, *p* = 0.0001, *r*^2^ = 0.37) (Fig. 1).

### SVM performance

An SVM model integrating HRV parameters and etiology was employed to differentiate PSH from noPSH events. The variables included SDNN, natural logarithms of the power spectral density (log PSD) of VLF, LF, and HF bands, the LF/HF ratio, and CI for both short- and long-time scales. Following feature selection based on a random forest algorithm, log PSD of VLF, log PSD of HF, and CIs were identified as key predictors distinguishing PSH from noPSH conditions.

Optimal SVM hyperparameters (*C* and gamma) were established by comprehensively comparing 100 different models, with *C* ranging between 0.5 and 10 and gamma between 0.01 and 10. The best-performing SVM model was configured with hyperparameters *C* = 9.96 and gamma = 2.56. The same hyperparameters were used to classify PSH and noPSH in the Pre_10_ and Pre_20_ conditions to avoid overfitting due to model adaptation to the data. The tenfold cross-validation test was used to validate our SVM model’s robustness.

During the event, the model classified PSH and noPSH conditions without misclassification in the training and validation tests. In the Pre_10_ condition (10 min before the event), the model showed perfect classification in the training phase and good performance in the validation phase, with a sensitivity of 67%, specificity of 100%, balanced accuracy of 83%, and *F*_1_-score of 80%. In the Pre_20_ condition, similar results were observed in the training phase, while the validation phase yielded a sensitivity of 100%, specificity of 33%, balanced accuracy of 68%, and *F*_1_-score of 67%.

Out of 100 tested models, 45 showed perfect classification in the training and validation phases. An additional 45 models had training error rates ranging between 2.3% and 9%, and only 10 models exhibited error rates around 12%. In the validation phase, only 3% of the models showed a misclassification rate of approximately 12% (Supplementary Material: Supplementary Fig. S1).

**Fig. 3:**
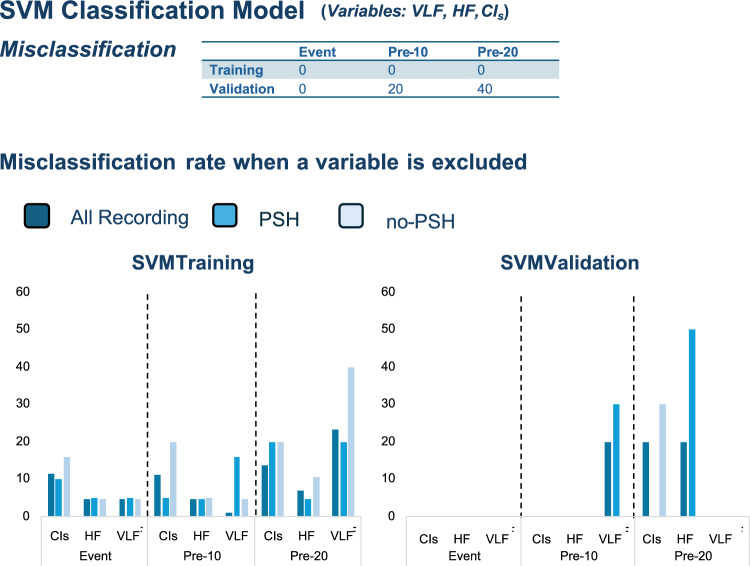
Support vector machine (SVM) results. SVM model’s performance (misclassification rate) when the complexity index (CI), the high frequency (HF), or the very low frequency (VLF) is excluded from the model. Dark blue represents the misclassification considering all PSH and noPSH conditions; blue represents the misclassification in PSH conditions; and light blue represents the misclassification of noPSH conditions

Upon assessing the influence of each variable through iterative exclusion, the CI_s_ significantly impacted classification accuracy, particularly during the events. During the event, eliminating CI_s_ from the model substantially increased the misclassification rate to 12% in the training test, while removing VLF or HF resulted in misclassification rates of 5%. Similar patterns were observed in the Pre_10_ and Pre_20_ conditions, highlighting the significant contribution of CIs to the model’s predictive accuracy. Detailed metrics and model fit measures are shown in Fig. 3 and Supplementary Material: Supplementary Fig. S1.

## Discussion

PSH remains a complex and largely unexplored phenomenon with significant implications for the management of patients with severe brain injuries. PSH worsens clinical outcomes, and currently, no quantitative biomarkers exist that accurately track or predict cardiovascular and ANS activities. To control PSH events, all patients received β-blocker therapy (propranolol or bisoprolol), antiepileptic prophylaxis (levetiracetam and/or phenobarbital), and antihypertensive agents (clonidine, amlodipine). Unlike noPSH events, during PSH, all patients showed a marked heart rate increase and distinct RR interval patterns with a characteristic tachogram shape (Fig. 2). These cardiac changes were accompanied by systolic blood pressure surges often reaching ≥ 180 mmHg, whereas respiratory rate remained within a range of 20 ± 5 breaths/min across all windows, with a transient peak around 30 breaths/min during PSH.

We observed a negative trend in the power of all HRV frequency bands for both PSH and noPSH, with a more substantial decrease in PSH alongside an increased VLF/(LF + HF) ratio, which increases more sharply in PSH. Furthermore, LF/HF increases only in PSH owing to a drop in HF power. These trends are coupled with reduced entropy (CI_s_ and CI_l_) that becomes evident 10 min before PSH and worsens during the event. Notably, these autonomic alterations occurred despite all patients being treated with β-blockers and antihypertensive agents specifically aimed at controlling PSH events.

Three principal theories consider the pathophysiology of PSH: the disconnection theory, the excitatory/inhibition ratio theory [1], and the neuroendocrine theory. The first two propose different mechanisms related to uncoupling of central nervous system (CNS) and ANS activity. The dissociation theory proposes that PSH results from a disconnection of the diencephalon and the upper brainstem, producing PSH as a “release” phenomenon. The excitatory/inhibition ratio theory posits that PSH arises from the impairment of descending inhibitory signals, which leads to the predominance of localized excitatory responses within the spinal cord, setting off amplified sympathetic activity. Interestingly, neuroendocrine theory, as exemplified by the study of Abdelhakiem et al. [6], suggests an etiology-independent (traumatic or nontraumatic) involvement of the pituitary axis in the progression of PSH. Considering the location of the pituitary and that the only connection to the hypothalamus is with the pituitary stalk, it was suggested that the trauma could impact the pituitary hormones [19, 20], such as in the release of corticotropin, correlated to the production of adrenocortical hormones essential for the organism to withstand the stressful situation [21, 22].

According to the Excitatory/Inhibitory Ratio (EIR) theory, it would be expected to observe increased markers of sympathetic activity, often interpreted as increased LF power in HRV, during PSH episodes. Contrary to this expectation, our analyses show a pronounced reduction in both LF and HF power in the pre-event window and during PSH, with a relatively larger decline in HF. This pattern is consistent with Eckberg’s critique of LF/HF interpretation: LF reflects mixed autonomic (and nonautonomic) influences, and increases in LF/HF frequently result from HF withdrawal (vagal loss) rather than from a surge in sympathetic outflow [23]. Consequently, LF/HF should not be interpreted as a direct measure of sympathovagal balance to avoid physiological misinterpretation. In parallel, the VLF/(LF + HF) ratio increased (Supplementary Material: Supplementary Fig. S3) and multiscale complexity (CIs) declined in the pre-event window, indicating slow-acting regulatory dominance and a loss of autonomic complexity, features that provide preonset information not reducible to heart rate alone.

The same consideration applies to disconnection theory. The increase in LF/HF observed during the PSH is due to a drop in the HF component and not to an increase in LF. However, the disconnection could be explained as an impaired central modulation of the sympathovagal system [24, 25].

On the contrary, the neuroendocrine theory highlights the role of hypothalamic–pituitary damage in PSH pathophysiology. Given that the hypothalamus plays a critical role in autonomic regulation, its impaired function might lead to a dominance of VLF activity, as our data suggest [26]. The hypothalamus plays a crucial role in both autonomic and neuroendocrine responses to stress. Injuring this area or its pituitary connections might result in high VLF levels, reflecting a reliance on less adaptive regulatory mechanisms [27, 28].

However, the VLF is also related to the renin–angiotensin–aldosterone system (RAAS) [29, 30], a hormone system that regulates blood pressure and cardiovascular function. The abnormal dominance of the VLF band in PSH could reflect a dysregulation of the RAAS, contributing to the complex cardiovascular manifestations observed in these patients [31]. Traditionally, RAAS has been associated with blood pressure regulation through endocrine actions, and its components have been identified in various organs, suggesting a broader influence on organ function [32]. The interaction between RAAS and the sympathetic nervous system has been well-documented [32, 33]. In stroke, dysregulation of the RAAS and the sympathetic nervous system can result in the abnormal release of hormones and neurotransmitters. This ultimately stimulates the hypothalamic–pituitary–adrenal (HPA) axis and increases cortisol secretion, which correlates with the stroke’s severity and damage to the insular cortex [34].

Nonetheless, the RAAS effect on the parasympathetic nervous system, especially in cardiac regulation, is less understood [35], and our analysis showed a significant drop in the HF component (expression of the parasympathetic system).

Angiotensin II (Ang II) receptors, particularly the AT1 receptor subtype, are distributed throughout the parasympathetic nervous system. They are present in the nodose ganglia, along the vagal nerve trunk, and at the terminals of vagal fibers in the heart [36]. These receptors can modulate vagal activity both at peripheral nerve endings and within central autonomic regions such as the nucleus tractus solitarii (NTS) and the dorsal motor nucleus of the vagus (DMV) [37].

Endogenous Ang II exerts a tonic inhibitory effect on cardiac vagal tone. Studies have shown that blocking AT1 receptors with antagonists such as losartan enhances vagal-induced bradycardia, indicating that Ang II normally suppresses vagal activity [38, 39].

Although circulating Ang II cannot cross the blood–brain barrier under normal conditions, it can influence central parasympathetic regulation via other mechanisms (Table 3). It attenuates baroreflex sensitivity by resetting the threshold at which baroreceptors respond to changes in blood pressure, leading to reduced vagal (parasympathetic) activity and increased sympathetic tone.

**Table 3:** Roles and mechanisms of angiotensin II in central autonomic regulation

Mechanism	Details	Effects/implications	References
Local synthesis	Ang II is locally synthesized in the brain Key areas: nucleus of the solitary tract (NTS) and dorsal motor nucleus of the vagus (DMV)	Regulation of autonomic functions such as heart rate and blood pressure	[40, 41]
Circumventricular organs	Ang II acts on regions lacking a blood–brain barrier, e.g., area postrema Influences neuronal activity affecting vagal output	Modulation of vagal activity and autonomic control	[42, 43]
Blood–brain barrier disruption	In hypertension, elevated Ang II increases blood–brain barrier permeability Allows Ang II to access central brain regions	Enhanced central actions of Ang II contribute to autonomic imbalance and hypertension	[44, 45]
Central nuclei effects	In the NTS: Microinjections of Ang II alter heart rate and blood pressure by affecting vagal tone May reduce baroreflex sensitivity via AT1 receptors Reactive oxygen species (ROS): Ang II increases ROS production in the NTS Impairs signaling to vagal motor neurons	In the NTS: Altered vagal tone and blood pressure regulation Decreased parasympathetic output ROS: Impaired baroreflex function	[46–48]
Baroreflex sensitivity	Ang II resets the threshold for baroreceptor response Leads to reduced vagal (parasympathetic) activity and enhanced sympathetic tone Exacerbates hypertension by diminishing baroreflex-mediated blood pressure regulation	Diminished ability to counteract elevated blood pressure Contribution to sustained hypertension	[49, 50]
Therapeutic interventions	Ang II receptor blockers (ARBs) and angiotensin-converting enzyme (ACE) inhibitors: Enhancing vagal tone: Block AT1 receptors, potentiating vagal activity and improving heart rate control Reducing central Ang II effects: Mitigate central actions of Ang II that cause autonomic imbalance in hypertension	Improved baroreflex sensitivity Enhanced parasympathetic function Reduced autonomic imbalance, aiding in hypertension management	[38, 51, 52]

NTS and DMV: These brain regions are critical for autonomic control, influencing heart rate, blood pressure, and other vital functions

Baroreflex: A feedback mechanism that helps maintain stable blood pressure by adjusting heart rate and vessel dilation in response to blood pressure changes

Autonomic imbalance: It refers to the disruption between the sympathetic and parasympathetic nervous systems, often leading to conditions such as hypertension

*Ang II* angiotensin II, *NTS* nucleus of the solitary tract, *DMV* dorsal motor nucleus of the vagus, *ROS* reactive oxygen species, *ARBs* angiotensin II receptor blockers

Finally, the reduction in the entropy, as observed in short- and long-time scales, might reflect not only the reduction in the brain–heart two-way interactions [53] but also a neuroendocrine system struggling to maintain homeostasis in the face of severe brain injury [54]. The reduction in entropy suggests a breakdown in the bidirectional interactions within the brain–heart axis. This leads to a less flexible and more deterministic autonomic output. Such changes are consistent with a system under stress or dysfunction, where normal variability and adaptability are compromised. It aligns with the notion of disrupted homeostasis due to RAAS dysregulation, as elevated levels of Ang II can inhibit parasympathetic activity and reduce baroreflex sensitivity, leading to a more rigid and less complex autonomic response [49, 50]. The RAAS, a key hormonal system involved in cardiovascular regulation, may contribute to this loss of complexity through its inhibitory effects on vagal tone and modulation of autonomic balance [35].

The patients in the study commonly exhibited brain damage in critical regions involved in autonomic regulation, such as the basal ganglia, corpus callosum (particularly the splenium), midbrain, and brainstem (Table 3). These damaged areas are integral to the functioning of the parasympathetic nervous system, particularly in modulating vagal tone and maintaining autonomic balance [25]. The RAAS plays a pivotal role in cardiovascular homeostasis by influencing these brain regions [35]. Damage to areas such as the midbrain and brainstem could disrupt RAAS-mediated modulation of the vagal system, potentially leading to impaired autonomic regulation and contributing to conditions such as PSH and hypertension observed in these patients.

Our findings suggest a significant role of the RAAS in mediating autonomic dysfunction following acquired brain injuries. While direct evidence linking RAAS activity to PSH is limited, several studies have demonstrated the influence of RAAS on autonomic regulation [33, 35, 50]. For instance, Ang II has been shown to inhibit parasympathetic activity via AT1 receptors in key autonomic regions such as the NTS and the DMV [35]. This inhibition could explain the observed decreased HF power and increased VLF/(LF + HF) during PSH episodes. These observations are consistent with a possible contribution of RAAS dysregulation to the abnormal VLF patterns in PSH. Nonetheless, VLF power is not a direct or specific marker of RAAS activity, and its interpretation requires caution. It should be regarded only as compatible with RAAS involvement, and confirmation would require concurrent biochemical measurements or interventional studies.

In the context of acquired brain injuries, hypothalamic or pituitary damage might disrupt normal RAAS function, contributing to the autonomic imbalance characteristic of PSH [55]. Although speculative, this proposed mechanism aligns with the neuroendocrine hypothesis of PSH and offers an expansion of the disconnection theory by incorporating aspects of hormonal regulation.

These findings could explain the effectiveness of the clinical use of propranolol (or equivalent) in managing PSH [56]. Propranolol is a nonselective beta-adrenergic receptor blocker that reduces sympathetic activity and inhibits renin release by blocking beta-1 adrenergic receptors in the juxtaglomerular cells of the kidneys [57, 58]. This inhibition leads to decreased renin secretion and, subsequently, lower levels of Ang II and aldosterone, attenuating their vasoconstrictive and sympathetic-enhancing effects [35]. By decreasing Ang II levels, propranolol may alleviate the inhibitory effect of Ang II vagal activity, thereby restoring vagal tone and improving autonomic balance [35, 59].

Our results contrast with the EIR model, which predicts increased sympathetic markers during PSH. Instead, significant LF and HF power reductions, with a relatively larger decline in HF, were observed, suggesting that PSH may not solely result from heightened sympathetic activity but may also involve parasympathetic withdrawal mediated by RAAS dysfunction.

By integrating the disconnection and HPA theories with the involvement of RAAS and considering the therapeutic effects of propranolol, a model where PSH results from both central autonomic disconnection and neuroendocrine dysregulation mediated by RAAS activity can be imagined.

While a rise in HR, along with increases in blood pressure, respiration, and sweating, is the most conspicuous and immediate manifestation of PSH and becomes evident only during the event, our aim was to investigate what additional information might be revealed by HRV analysis. In our dataset, HF power declined markedly in the pre-event window, LF power also fell, and the very-low-frequency component became relatively predominant (expressed as an increased VLF/[LF + HF] ratio; Supplementary Material: Supplementary Fig. S3 and Supplementary Table S3). At the same time, multiscale complexity indices (CIs) progressively decreased, reflecting a decomplexification of the brain–heart interaction. VLF, HF, and CIs were the features driving the model’s preonset discriminative performance. Taken together, these findings indicate that HRV-derived measures provide anticipatory and orthogonal information to HR: Slow regulatory dominance (VLF) and loss of autonomic complexity (CIs) emerge before the clinical onset and can signal an evolving PSH episode earlier than tachycardia alone. This complementary information may be valuable both for early detection and for mechanistic hypotheses on slower regulatory processes that precede the overt autonomic storm.

Our findings suggest that the VLF and HF power and the complexity index in short-time scales contribute to differentiating PSH and noPSH. Using the SVM classification model demonstrates the potential of machine learning algorithms in classifying the PSH event. Moreover, these characteristics differentiate them 10 and 20 min before the event.

Nonetheless, the physiological mechanisms underlying the VLF component in HRV remain not fully understood, and its relationship with the RAAS is not extensively explored in literature. While some studies have suggested that the VLF band may reflect hormonal influences and slower-acting regulatory mechanisms, including those mediated by the RAAS [29, 60], direct evidence linking increased VLF power to RAAS activity is limited.

Our observation of an abnormal dominance of the VLF band before PSH episodes in these patients could indicate a multifactorial dysregulation of slow-acting autonomic mechanisms, possibly involving neuroendocrine pathways such as the RAAS. However, given that VLF power is influenced by multiple factors (including thermoregulatory and vasomotor activity, endothelial factors, baroreflex modulation, and hormonal fluctuations [29, 30, 61–64]) and decreased in absolute terms during the events, its interpretation should be approached with caution. Future studies combining HRV analysis with direct measurements of RAAS components could clarify this relationship. Moreover, exploring the impact of autonomic-targeted or sympatholytic interventions on PSH symptoms could help identify more effective therapeutic strategies.

Nevertheless, early detection of PSH through our HRV/SVM model could significantly reduce complications such as hyperthermia and spasticity, enhance recovery trajectories, and alleviate intensive care unit (ICU) burden by optimizing resource utilization. For instance, our data indicate that timely interventions based on predictive analytics may prevent harmful physiological sequelae, thereby improving patient outcomes.

This study presents several important limitations. The primary limitation is the sample size, as 24 PSH events were recorded in six patients and compared with similar conditions characterized by increased cardiac activity. The limited number of recorded PSH episodes reflects the inherent challenge of identifying the precise onset of PSH. Moreover, the timing of PSH was based on the PSH assessment measure [14] and determined by clinical staff, which may introduce bias. Again, in our sample, four out of six patients presented with predominant right-hemisphere lesions. However, the locations were heterogeneous, involving occipital, temporal, thalamic, and brainstem structures, rather than a consistent cortical or subcortical region. Therefore, while lateralization of autonomic control has been described, particularly with right insular and fronto-temporal involvement [65–67], our small and heterogeneous cohort does not allow conclusions regarding hemispheric dominance. This aspect deserves a dedicated investigation into larger and more anatomically homogeneous populations.

Despite this limitation, the study provides significant insight into PSH. All patients exhibited consistent autonomic patterns at PSH onset, regardless of etiology. Moreover, this nominal sample of subjects and events far exceeds other reports of the phenomena.

This study provides new insights into the underexplored influence of RAAS on the VLF component of HRV, particularly in the context of PSH. Future research incorporating direct measurements of RAAS activity alongside HRV analysis could further elucidate this relationship and enhance the understanding of PSH pathophysiology.

By integrating HRV analysis with machine learning, a predictive model was developed to identify PSH episodes up to 10 min before clinical onset. This capability may enable timely interventions to reduce the severity and duration of PSH episodes in critically ill patients.

The findings suggest targeted biochemical assessments to improve clinical management. Monitoring circulating renin, angiotensin II, and aldosterone levels could clarify RAAS dysregulation in PSH, while cortisol assessment may provide insights into concurrent HPA axis activation.

The predictive model may be integrated into standard monitoring workflows to support early PSH detection. Its application in future clinical trials could help evaluate RAAS-targeted therapies and improve patient outcomes. These findings enhance mechanistic understanding and present direct clinical implications, offering new opportunities for critical care teams to optimize PSH management.

## Supplementary Information

Below is the link to the electronic supplementary material.

Supplementary file1 (DOCX 1093 KB)

## Data Availability

The datasets generated and/or analyzed during the current study are not publicly available owing to privacy/ethical restrictions but are available from the corresponding author on reasonable request.
