# Supplementary material for: New insights and predictability from in vivo recordings of paroxysmal sympathetic hyperactivity in disorders of consciousness
Source: Clin Auton Res. 2025 Dec 24;36(2):175–90. doi: 10.1007/s10286-025-01175-z (PMC13068689; doi:10.1007/s10286-025-01175-z)
Supplement: Supplementary file 1 — Supplementary file1 (DOCX 1093 KB) [file 10286_2025_1175_MOESM1_ESM.docx]

**New Insights and Predictability from In-Vivo Recordings of Paroxysmal Sympathetic Hyperactivity in Disorders of Consciousness**

Supplementary Material


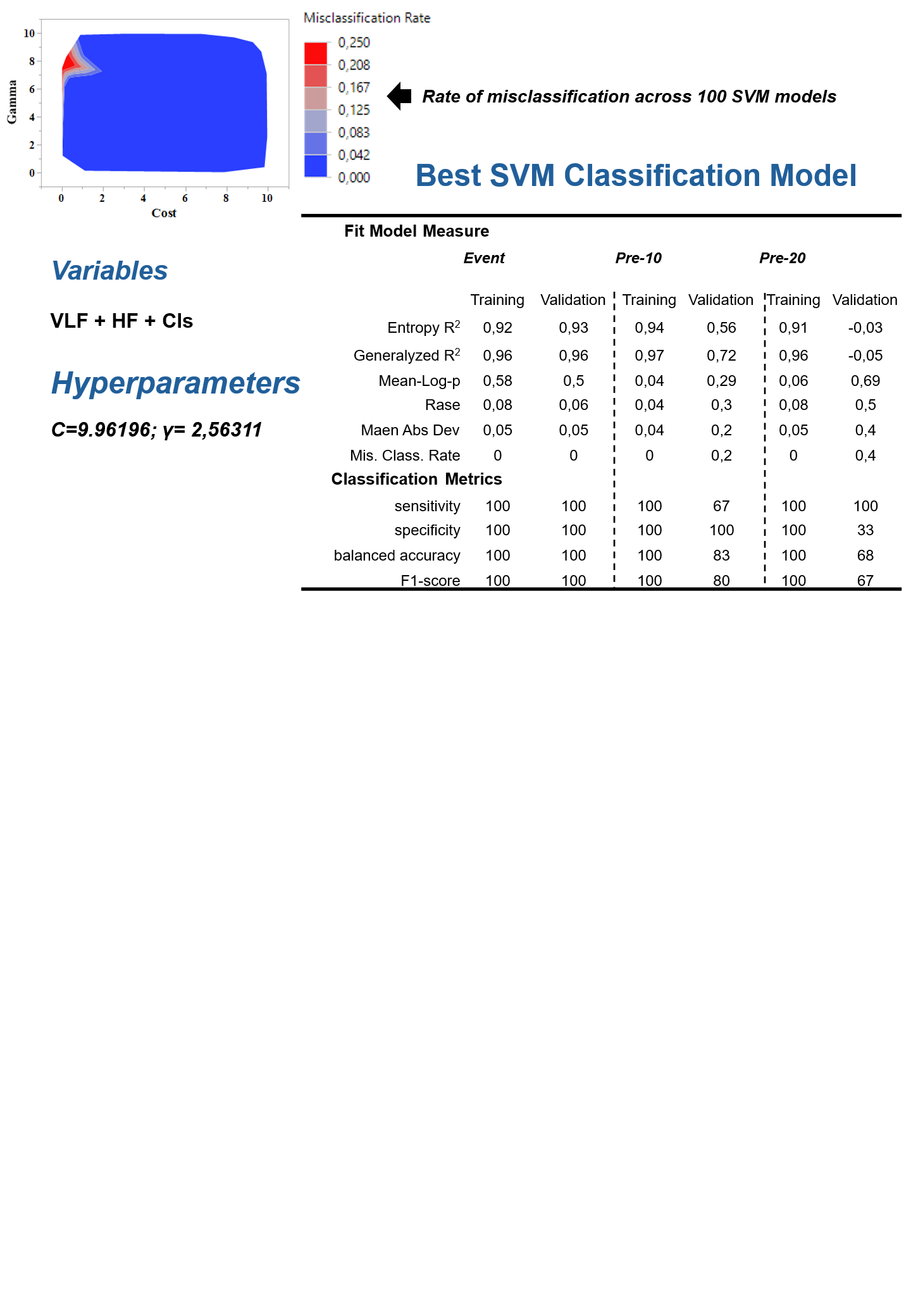


Figure s1: Support vector machine (SVM) results: SVM PSH/noPSH classification model. On the left corner, the performance results of 100 SVM models based on C and γ SVM hyperparameters. C and γ are the SVM hyperparameters used. For the event, pre10 and Pre20, in training and 10-fold cross-validation tests, are provided the Entropy R2 (goodness-of-fit measure for classification models. It is based on the concept of entropy, which represents the uncertainty in a dataset), Generalized R2 (measures the proportion of variance in the dependent variable explained by the model. It is an extension of the traditional R2 used for linear regression and can be applied to non-linear models like SVM). Mean-log p (Mean Negative Log Likelihood- measures how well the predicted probabilities from the SVM model match the actual outcomes), RASE (Root Average Squared Error - measures the average squared difference between the predicted and actual values), Mean Absolute Deviation (average of the absolute differences between the predicted and actual values). Considering the true positive (TP) and false positive (FP) classifications, sensitivity (TP/(TP + FN)) and specificity (TN/(TN + FP)) indicate how well the model identifies positive and negative cases respectively, balanced accuracy ((Sensitivity + Specificity)/2) is the average of sensitivity and specificity; F1 score (2*(precision*sensitivity)/(precision + sensitivity)) measures the model’s accuracy, combining both precision and recall into a single metric, where precision (TP/(TP + FP)) is the ratio between true positives and the sum of true positives and false positives.


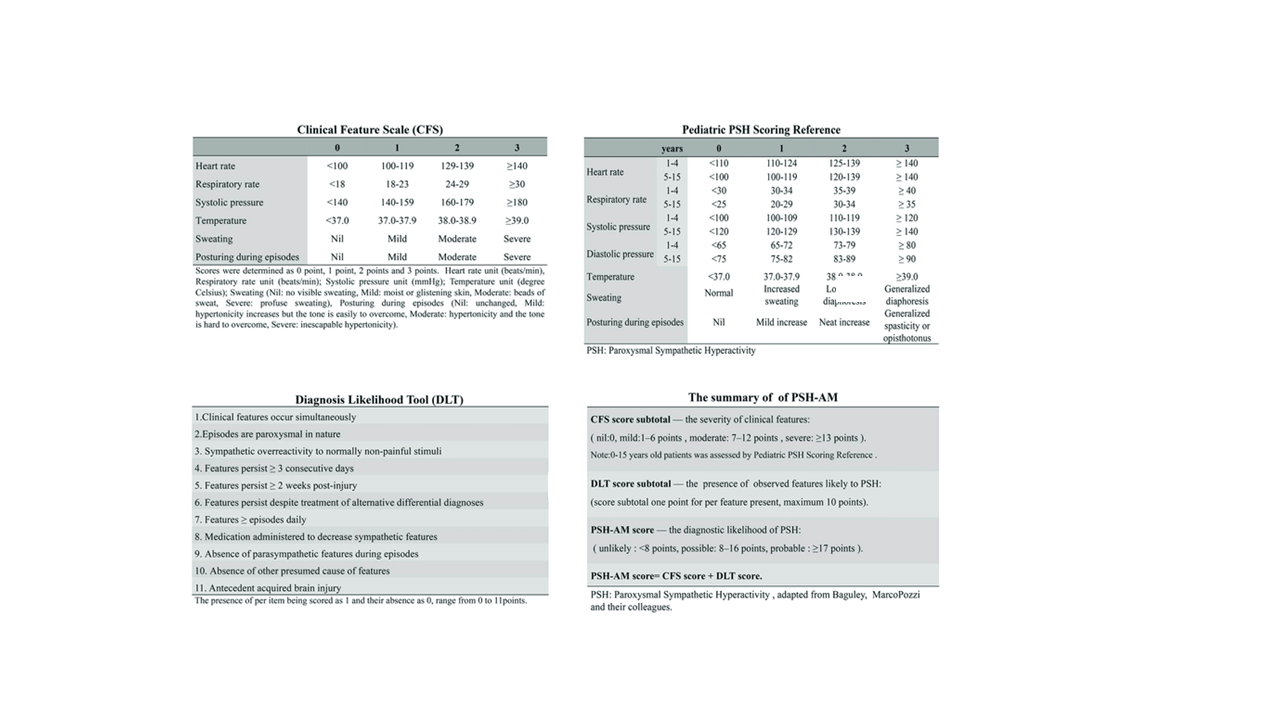


Figure s2: Scales for PSH assessment


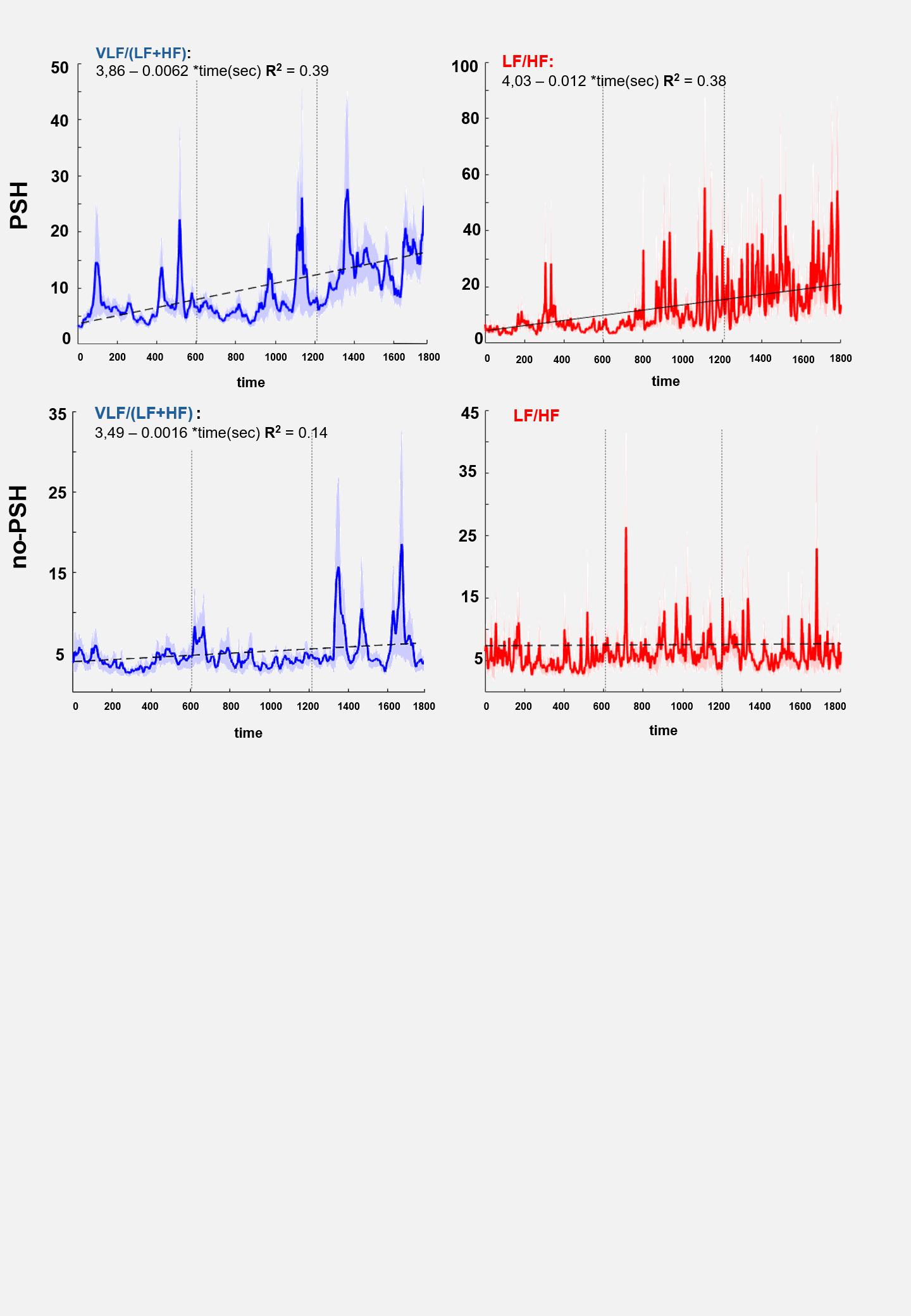


Figure s3: Autonomic Ratio. The rows are the PSH and noPSH conditions, respectively. The vertical dashed lines represent the division of 1800 seconds (30 minutes) into three equal blocks of 600 seconds (10 minutes.). In blue and red are the VLF/(LF+HF) and LF/HF ratios along time, with the relative linear trends, respectively. No linear trend was observed for the LF/HF ratio in the noPSH event. Conversely, the VLF/(LF+HF) ratio showed a significant increasing trend over time in both conditions, with a greater rate of increase in the PSH condition. The LF/HF ratio demonstrated a significant positive trend only in the PSH condition. LF power reflects mixed autonomic and non-autonomic inputs and increases in LF/HF commonly result from HF (vagal) withdrawal rather than true sympathetic activation; therefore, LF/HF should not be interpreted as a direct index of sympathovagal balance.

Table s1: Respiration in breaths*min^-1^ in the different time recording windows

| **Condition** | **window** | **mean** | **Median [IQR]** | **Min-max** |
| --- | --- | --- | --- | --- |
| **PSH** | T20 | 19±5 | 19 [9.6] | 12-27 |
|  | T10 | 19±5 | 19 [6.3] | 12-30 |
|  | Event | 21±4 | 21 [6.9] | 13-31 |
| **no-PSH** | T20 | 18±6 | 15 [7.8] | 12-31 |
|  | T10 | 19±6 | 16 [6] | 12-31 |
|  | Event | 19±6 | 16 [6.9] | 12-30 |

Table s2: Systolic and Diastolic pressure in mmHg in the different time recording windows

|  |  | **Systolic** | | | **Diastolic** | | |
| --- | --- | --- | --- | --- | --- | --- | --- |
| **Condition** | window | mean | Median [IQR] | Min-max | mean | Median [IQR] | Min-max |
| **PSH** | Before event | 136±9 | 137 [13.7] | 122-155 | 82±10 | 80 [13.7] | 61-101 |
|  | Event | 183±5 | 182 [7.5] | 175-198 | 92±8 | 91 [12.5] | 78-105 |
| **no-PSH** | Before event | 115±7 | 115 [10] | 100-137 | 69±7 | 70 [13.5] | 60-80 |
|  | Event | 125±8 | 125 [9.5] | 110-150 | 73±5 | 72 [6.5] | 67-82 |

Table s3: Regression Analysis of PSD Components Over Time for PSH and noPSH Conditions

| Component | Condition | Intercept | SE Intercept | Time Coefficient (per sec) | SE Time Coefficient | F-Ratio | p-value | R² | 95% CI for Time Coefficient |
| --- | --- | --- | --- | --- | --- | --- | --- | --- | --- |
| LF/HF Ratio | PSH | 4.03 | 0.37 | 0.012 | 0.0004 | 1121.80 | <0.0001 | 0.38 | [0.0112, 0.0126] |
|  | noPSH | 8.63 | 0.16 | 0.0002 | 0.0002 | 2.14 | 0.10 | 0.001 | [-0.00008, 0.0006] |
| VLF/(LF+HF) | PSH | 3.86 | 0.19 | 0.0062 | 0.0002 | 1160.40 | <0.0001 | 0.39 | [0.0058, 0.0066] |
|  | noPSH | 3.49 | 0.10 | 0.0016 | 9.82e-5 | 283.40 | <0.0001 | 0.14 | [0.0014, 0.0018] |
| - PSH: paroxysmal sympathetic hyperactivity condition. - noPSH: non-psh condition. - Intercept: estimated psd value at time zero. - Time Coefficient: estimated change in PSD per second. - SE: standard error of the coefficient. - F-Ratio: ratio used in ANOVA to determine the overall significance of the model. - p-value: indicates the statistical significance of the coefficient. - R²: coefficient of determination, representing the proportion of variance explained by the model. - 95% CI: for time coefficient: confidence interval for the time coefficient, providing a range of values within which the true coefficient is likely to fall. | | | | | | | | | |
